# CLINICAL AND FUNCTIONAL OUTCOMES OF AQUATIC PLYOMETRICS IN ADOLESCENT PATELLOFEMORAL PAIN SYNDROME: A DUAL-ARM RANDOMIZED CONTROLLED TRIAL

**DOI:** 10.2340/jrm.v58.46495

**Published:** 2026-10-19

**Authors:** Mazyad A. ALOTAIBI, Ragab K. ELNAGGAR, Ahmed S. ALHOWIMEL, Saud M. ALRAWAILI, Tamer E. ELNEGAMY, Hesham A. ALFEHEID, Maged A. BASHA, FatmaAlzahraa H. KAMEL, Saleh M. ALORAINI, Feras ALSULTAN, Amal ALHARBI, Mahmoud S. ELFAKHARANY

**Affiliations:** 1 Department of Health and Rehabilitation Sciences, Prince Sattam bin Abdulaziz University, Al-Kharj, Saudi Arabia; 2 Department of Physical Therapy, College of Applied Medical Sciences, Qassim University, Buraydah, Saudi Arabia; 3 Department of Physical Therapy for Surgery, Faculty of Physical Therapy, Cairo University, Giza, Egypt; 4 Department of Physical Therapy for Pediatrics, Faculty of Physical Therapy, Cairo University, Giza, Egypt

**Keywords:** patellofemoral pain syndrome, aquatic therapy, plyometric exercise, muscle strength, disability, health-related quality of life

## Abstract

**Objective:**

Optimal management of adolescent patellofemoral pain syndrome requires evidence-based interventions that maximize physical recovery without overloading the patellofemoral joint. This study investigated the efficacy of aquatic plyometric (Aquatic-Plyo) exercises on pain, muscle strength, and functional disability and health-related quality of life in this demographic.

**Design:**

A dual-arm randomized controlled trial.

**Subjects/Patients:**

56 adolescents with patellofemoral pain syndrome (age: 10–18 years).

**Methods:**

Adolescents were allocated to an experimental Aquatic-Plyo group or a control group performing conventional exercises. Both arms completed 45-min sessions twice weekly for 12 weeks. Pre- and post-treatment assessments included pain intensity, isokinetic knee (quadriceps and hamstrings) torque at 120°/s and 180°/s, functional disability, and health-related quality of life.

**Results:**

Mixed-model ANOVA confirmed the therapeutic dominance of the Aquatic-Plyo protocol. It yielded superior pain relief (*p* = 0.004, η^2^p = 0.14) and larger increases in quadriceps [120°/s (*p* = 0.006, η^2^p = 0.13); 180°/s (*p* = 0.008, η^2^p = 0.12)] and hamstring [120°/s (*p* = 0.007, η^2^p = 0.13); 180°/s (*p* = 0.023, η^2^p = 0.09)] peak torque. Moreover, the Aquatic-Plyo intervention drove greater reduction in functional disability (*p* = 0.03, η^2^p = 0.08) and notable enhancement in perceived health-related quality of life (*p* = 0.023, η^2^p = 0.09).

**Conclusion:**

Aquatic-Plyo exercises effectively reduces pain and disability while improving muscle strength and health-related quality of life in adolescents with patellofemoral pain syndrome, making it a valuable addition to conservative rehabilitation strategies.

## LAY ABSTRACT

Patellofemoral pain syndrome frequently compromises physical function and health-related quality of life in adolescents, necessitating refined conservative rehabilitation protocols. This randomized controlled trial compared aquatic plyometric exercises with standard physical therapy. Following 12 weeks of treatment, the aquatic-plyometric cohort exhibited clinically significant advantages. They experienced superior pain relief and greater attenuation of functional disability. From a musculoskeletal perspective, the aquatic-plyometric group demonstrated enhanced isokinetic strength of the knee flexors and extensors, validating the protocol’s ability to safely build muscle torque. Importantly, these physiological gains translated into meaningful improvements in perceived health-related quality of life. For clinicians seeking to balance joint preservation with robust muscular recovery, aquatic plyometrics represent a highly efficacious, evidence-based modality for managing adolescent patellofemoral pain syndrome.

The most prevalent knee pain condition in adolescents is patellofemoral pain syndrome (PFPS), commonly identified by pain that is centred on the patella. This pain typically stems from excessive strain during knee flexion and extension movements. This condition can be exacerbated by activities that are non-traumatic in nature, such as squatting, climbing up and down stairs, running, and jumping (1). The occurrence of PFPS is particularly pronounced in adolescents within the age bracket 12 to 17 years and is more frequently seen in physically active individuals (2). Females in this demographic experience PFPS at a rate of 1.5 to 3 times greater than their male counterparts (3, 4). The onset of PFPS can be attributed to several factors. One significant factor is abnormal patellar tracking, wherein the patella deviates from its normal trajectory within the femoral groove. This can be a consequence of muscular imbalance, including quadriceps weakness, hamstring tightness, delayed activation of vastus medialis obliquus, and insufficient hip adductors and lateral rotators strength, which can compromise patellar alignment and stability (5, 6). Other factors that play a role in the development of PFPS are overuse and repetitive stresses on the knee, particularly from activities like excessive running or jumping, leading to patellar tendon irritation and inflammation (7). Furthermore, biomechanical issues like flat feet and incorrect foot mechanics can influence the forces that the knee joint experiences, therefore heightening the risk of PFPS (5).

The PFPS poses various challenges to adolescents, typically resulting in peri- or retropatellar pain that significantly limits their ability to participate in physical activities and daily routines (4). This pain often interferes with their performance in sports and other physical pursuits due to concerns about exacerbating their condition. So, they may gravitate towards a more sedentary lifestyle, which can adversely affect their fitness and overall health. Beyond the physical pain, PFPS can undermine the quality of life for adolescents by restricting their involvement in social and recreational activities, potentially influencing their mood and self-esteem (8). Moreover, adolescents with PFPS may exhibit muscle weakness and altered movement patterns, which can exacerbate their pain and increase their risk of injury (9). This consequently may highlight the pressing need for developing more refined treatment approaches that can effectively address the unique challenges that adolescents with PFPS encounter.

The literature has underscored the significant role of physical rehabilitation strategies for adolescents experiencing PFPS, suggesting that these approaches are as vital as medical interventions in mitigating impairments and enhancing treatment outcomes (10). Exercise is central to these rehabilitation efforts, with a range of exercise paradigms being explored for this patient population (11–13). However, the varying success rates among these programmes have led to continued uncertainty regarding which exercise intervention should take precedence (14). Plyometric training, also referred to as stretch-shortening cycle training, is a dynamic exercise approach that focuses on explosive movements to bolster strength, power, and speed. This approach typically involves a sequence of eccentric actions (muscle lengthening) that are promptly succeeded by concentric actions (muscle shortening), designed to maximize the rate of force development (i.e., generating a substantial force in minimal time) (15). Earlier investigations have shown that such a training approach is safe, practical, and effective in enhancing numerous aspects of motor function among children/adolescents with diverse physical impairments (16–21).

Aquatic Plyometrics (Aquatic-Plyo), incorporating plyometric exercises within an aquatic setting, has emerged as a prominent rehabilitation strategy for children and adolescents with musculoskeletal and neuromotor disorders (22–26). The Aquatic-Plyo approach offers several distinctive advantages over standard land-based training. By harnessing the upthrust buoyant force of water, Aquatic-Plyo significantly mitigates the weightbearing stresses and impact forces by 33–62%, contingent upon performance speed and immersion depth, which effectively help minimize injury risks and delay fatigue onset (27). Additionally, Aquatic-Plyo allows for the implementation of more demanding exercise, leveraging the resistance associated with water viscosity and other dynamic qualities like wave drag and surface profile (28). Furthermore, because adolescents typically perceive aquatic activities positively, Aquatic-Plyo can foster their interest and promote a longer and more consistent training schedule (22–25). While Aquatic-Plyo is likely to confer multiple benefits, there is currently a lack of evidence supporting its efficacy for adolescents with PFPS. Accordingly, this study was designed to investigate the efficacy of Aquatic-Plyo compared with standard practice (defined as a conventional land-based physical therapy programme encompassing strengthening, flexibility, and proprioceptive exercises) on pain, muscle strength, disability, and HRQoL in adolescents with PFPS. We hypothesize that Aquatic-Plyo will produce more favourable effects on these rehabilitation outcomes than the standard exercise regimen.

## MATERIALS AND METHODS

### Design and ethics

The study was designed as a prospective randomized controlled trial with a single-blind protocol, wherein the outcome evaluator remained unaware of the intervention allocations. The study was conducted over the period from June 2023 to May 2024 at the physical therapy outpatient clinic at Prince Sattam bin Abdulaziz University (PSAU). Prior to participants’ recruitment, the study protocol (No: RHPT/0023/0019) was reviewed and sanctioned by the Physical Therapy Research Ethics Committee at PSAU, ensuring compliance with the ethical guidelines of the Declaration of Helsinki 1975. Participants, along with their legally authorized representatives, were mandated to sign an informed consent after receiving a thorough explanation of the study’s objectives, potential benefits, and associated risks. The study protocol was also registered at ClinicalTrials.gov with the assigned identification number (NCT07679464).

### Participants

The study included 56 adolescents diagnosed with PFPS, using the ICD classification system, which addresses issues related to body structure, function, activity, and participation (ICD-10 code for PFPS: M22.2X9) (29). Eligible participants were aged 10 to 18 and reported anterior, peri- or retropatellar pain exceeding 3 on the visual analogue scale for a duration of over 3 months. Their pain worsened during activities such as prolonged sitting, kneeling, squatting, hopping, running, or stair climbing, yet improved with rest. They experienced a gradual onset of symptoms and exhibited pain upon palpation of the patellar facet, during double-legged squats, or while descending from a 25-cm step, as well as positive findings from Clarke’s sign and the McConnel test (30). Participants also exhibited a minimum strength of grade 3, “fair,” in their proximal lower extremity muscles – that is, they were capable of executing movements against gravity through the full range of motion without external resistance and effectively holding the contraction (31). Participants were not eligible if they had undergone knee surgery, suffered recent trauma, or exhibited any intra-articular issues. Those with patellar subluxation or dislocation were also excluded, as were those who had taken anti-inflammatory medications or corticosteroids in the 6 months leading up to the study.

### Randomization and group assignment

To promote the balance of participants’ characteristics across the two groups (control and Aquatic-Plyo), a stratified randomization strategy was implemented. Participants were stratified into 4 age- and gender-specific subgroups: male and female adolescents aged 10–14 and > 14–18 (*n* = 16, 20, 8, and 12, respectively). The 14-year age threshold was selected to roughly distinguish early/mid-puberty from late puberty, ensuring that pubertal-related variations in muscle development and biomechanics were equally distributed. These strata were utilized exclusively to prevent accidental baseline imbalances between the 2 main treatment arms during allocation, rather than for subsequent statistical subgroup analyses. A randomization sequence was generated for each subgroup by an independent researcher using a web-based random generator (GraphPad^®^, QuikCalcs; https://www.graphpad.com/quickcalcs/randomN1/), with the sequence kept hidden from investigators to ensure blinding. Participants were assigned to the study groups according to these sequences, beginning with the first participant and alternating between the control and Aquatic-Plyo groups until all participants were assigned.

### Outcome measures

The assessments were performed by the same evaluator, who was blind to the group assignments and not involved in administering the treatment or analysing data. These assessments were conducted at the baseline and upon completion of the 12-week intervention programme. The primary metrics assessed were pain intensity and muscle strength, while disability index and HRQoL were designated as secondary metrics.

### Pain intensity

Participants were prompted to report the level of pain they experienced over the past week using the Numeric Pain Rating Scale (NPRS). Both resting and movement pain were considered as a single construct, given their intertwined effect on disability and adaptive functioning (32). The NPRS operates on a self-reported basis, featuring an 11-point scale from 0 to 10. It is defined by verbal cues, with 0 indicating “no pain,” 5 representing “moderate pain,” and 10 signifying “very severe pain.” Participants were instructed to indicate the number that best reflects the intensity of knee pain they encountered. The NPRS is well established, extensively utilized, and has proven reliable for measuring pain intensity in children/adolescents with acute or chronic musculoskeletal conditions (22, 33, 34).

### Muscle strength

Using the HUMAC NORM isokinetic dynamometer (Humac Norm 2009, CSMi, Stoughton, MA, USA), the peak torque of quadriceps and hamstrings was during concentric movements. This assessment followed the prescribed protocol established by the manufacturer for assessing lower extremity strength. The dynamometer was calibrated before testing, after which participants engaged in a warm-up consisting of 10 knee flexion–extension movements at a speed of 240^o^/sec, exerting submaximal effort. Peak torque measurements were taken at speeds of 120^o^/sec and 180^o^/sec across a knee flexion range of 0–90^o^. Participants were seated in a customizable ergonomically designed dynamometer chair, which featured a backrest tilted 10^o^ backward from the vertical orientation. To ensure stability and prevent the recruitment of non-target muscles, Velcro straps were securely fastened around the thigh, waist, and upper torso. The dynamometer’s axis of rotation was precisely aligned with the anatomical axis of the knee, enhancing the measurement accuracy and reliability. The resistance arm was installed on the distal end of the lever arm of the dynamometer and attached to the leg (3 cm above the malleoli) with a posterior Velcro strap. Participants were instructed verbally to give their best effort during the test and received real-time visual feedback on a screen displaying the torque development curve. Each muscle was tested in 3 trials, interspersed with 2-min rest intervals. In each trial, 3 maximal contractions were performed in succession, with the quadriceps and hamstrings assessed in the isolated concentric knee extension and flexion modes, respectively. The average torque measurements, expressed in Newton-metres (Nm), were computed.

### Disability

The extent of disability and functional limitations was measured using the Patellofemoral Disability Index (PFDI). This index has proven to be a valid and feasible assessment tool, providing reliable and accurate measurements of disability related to PFPS (35). With 10 specific items, the PFDI delivers a comprehensive evaluation of physical capabilities. It assesses performance along a continuum of activities, including high-demand activities such as running and jumping, as well as routine tasks like sitting, kneeling, squatting, walking, limping, and stair navigation. It further addresses the sense of instability. The PFDI employs a 6-point rating scale from 0 to 5 for each item, where 0 signifies no disability and 5 corresponds to being bed-bound or experiencing intensified symptoms. The index captures varying levels of disability: minimal (1), minimal-to-moderate (2), moderate (3), and severe disability (4). A score of 50 is possible, with larger scores indicating a higher degree of disability.

### Health-Related Quality of Life

To assess HRQoL, the Arabic version of the validated MOS 36-Item Short Form Health Survey (SF-36) was used (36). This instrument features a collection of generic, coherent, and user-friendly HRQoL measures that rely on self-reported information. The SF-36 addresses 8 health domains: physical functioning, bodily pain, limitation in roles due to physical health, limitations arising from personal or emotional difficulties, social functioning, emotional well-being, energy and fatigue, and overall health perception. In addition, the SF-36 features a single item that captures the perceived change in health status. Each item is rated on a scale of 0 to 100, representing a continuum from the lowest to the highest HRQoL. Scores for items within each health domain are summed and averaged to derive the scores for the 8 domains. The total score is calculated by averaging all the items completed by the respondents.

### Interventions

*Aquatic plyometrics.* The Aquatic-Plyo training was conducted in a 3x4 water pool under the guidance of a trained physical therapist, following performance and safety recommendations set by the National Strength and Conditioning Association (37). It was lower-body-focused, 45 min long, and repeated twice a week, ensuring a minimum recovery interval of 48 h, for a duration of 12 weeks. Immersion was at waist level, with the water temperature held between 30^o^ C (86^o^ F) and 31^o^ C (88^o^ F) and the room temperature adjusted within a range from 26^o^ C (78^o^ F) to 28^o^ C (82^o^ F). A progressive approach to Aquatic-Plyo training was implemented to introduce a new training stimulus, minimize fatigue or injury risks, and avoid adaptation. The programme commenced with low-volume sessions in the first 4 weeks, progressively increasing the number of sets and repetitions once from week 5 to 8 with a second increase from week 9 to 12.

The training, in each session, began with a general warm-up (10 min) that featured flexibility exercise, light cardiorespiratory exercise (such as treadmill walking or stationary biking, as favoured) performed at an intensity of no more than 50% of the age-predicted maximum heart rate, synchronized arm movements coordinated with breathing, as well as water-based free jogging. Subsequently, participants engaged in 10 lower body Aquatic-Plyo exercises for around 25 to 30 min, replicating the exact methods outlined in previous research. The full specification, training volume, guidelines for performance, and progression rates for these exercises are included in Table SI. To wind down, a 5-min cool-down was implemented, comprising self-paced walking and static stretching.

*Standard exercises.* The control group participants engaged in a standard exercise regimen that lasted approximately 45 min, overseen by a paediatric physical therapist with more than 10 years of professional experience. The session began with a warm-up that included 5 min of stationary biking or elliptical workouts, along with dynamic stretches targeting hip flexors, quadriceps, hamstrings, and calves. The session concluded with a cool-down period featuring 5 min of light cycling or walking, followed by lower body-focused static stretches. The core exercise segment spanned 35 min and comprised the following:

1. Strengthening components (15 min): The exercises targeted quadriceps strength with isometric wall sits, short-arch knee extensions, and elastic bands-resisted knee raises. It also targeted the hip and gluteal muscles through bridges combined with leg extensions, clamshells, and lateral band walks, as well as core stabilization via front, side, and reverse blanks.
2. Flexibility components (10 min): The exercises involved patellar mobilization, static stretching for hamstrings, quadriceps, and iliotibial band, along with foam rolling to relieve muscle tightness and/or soreness.
3. Proprioception and balance components (10 min): The training included single-leg balance that transitioned from stable to unstable surfaces, perturbation exercises (on a wobble cushion and balance board), agility drills (such as forward–backward hops and lateral shuffles), and plyometric workout featuring mini or low-height jumps.

### Sample size estimation

To avert the risk of a false negative conclusion regarding the intervention’s efficacy, *a priori* sample size estimation was performed using PASS software version 16.0.1 (NCSS, Kaysville, UT, USA) based on an F-test for the group-by-time interaction in a repeated-measures analysis of variance (ANOVA). The analysis was structured as a 2-by-2 mixed model design, with 2 groups (control and Aquatic-Plyo), each assessed at 2 intervals (pre- and post-intervention). The primary intention, in this case, was to analyse the difference in pain intensity changes from pre- to post-intervention among groups (i.e., the group-by-time interaction effect), as pain is the most prevalent and functionally limiting symptom of PFPS driving care-seeking behaviour in adolescents. A total sample size of 44 participants (22 per group) was determined to achieve 90% power for detecting a mean change difference of 2.9 in pain intensity. This determination was based on preliminary data derived from an internal pilot study involving 10 adolescents with PFPS (5 per group), indicating a standard deviation of 1.9 before the intervention and 3.1 afterwards, a correlation of 0.410 between measurements, and a significance level of 0.05. To accommodate an anticipated dropout rate of approximately 20%, an additional 12 participants were included, resulting in a total of 56 participants (28 in each group).

### Statistical analysis

To adhere to the intention-to-treat principle, all randomized participants were included in the final analysis. Missing follow-up data (*n* = 3) were assumed to be missing at random, as dropouts were related to scheduling and personal issues rather than treatment efficacy. To handle missingness without underestimating standard errors or introducing bias, we utilized multiple imputation. Five imputed datasets were generated using multivariate regression equations, and the results were pooled to properly account for imputation uncertainty. The assumption of normality was verified using the Shapiro–Wilk test, which confirmed the normal distribution of data for muscle strength, disability, and HRQoL (*p* > 0.05). However, pain intensity data (NPRS scores) deviated significantly from normality (*p* < 0.05). Accordingly, a 2-by-2 mixed-model ANOVA was employed to analyse the differences in pre-to-post changes (i.e., the group-by-time interaction effect) among the study groups regarding all outcome measures, excluding pain, for which the aligned ranks transformation ANOVA was applied, as it is highly robust against normality violations and specifically designed for evaluating interaction effects in non-parametric data. To characterize the pre-to-post intervention changes within each individual group, exploratory paired-sample *t*-tests were utilized. These *t*-tests were applied strictly conditionally, only when the omnibus ANOVA yielded a significant interaction effect. Because this significant omnibus test acts as a gatekeeper that protects the overall family-wise error rate, no additional adjustments to the *p*-value were deemed necessary for these subsequent within-group comparisons. The formula for Hedges’ *g* and eta-squared partial (*η*^2^p) were utilized to compute effect sizes for the within-group changes and between-group differences, respectively. The analyses were carried out using Stata software, v15.1 (Stata Corp, College Station, TX, USA), and graphs generation was done employing GraphPad Prism, v10.3.0 (GraphPad Software Inc, San Diego, CA, USA). A significance level of < 0.05 was adopted across all tests.

## RESULTS

### Participants flow and retention

The CONSORT flow diagram in Fig. 1 illustrates the recruitment and retention of participants throughout the study. Of 87 adolescents screened for PFPS, 56 were eligible to proceed to randomization. Of these, 3 participants – 2 from the control group and 1 from the Aquatic-Plyo group – could not complete the assigned intervention or were lost during the follow-up, primarily due to scheduling conflicts or unrevealed personal issues.

**Fig. 1:**
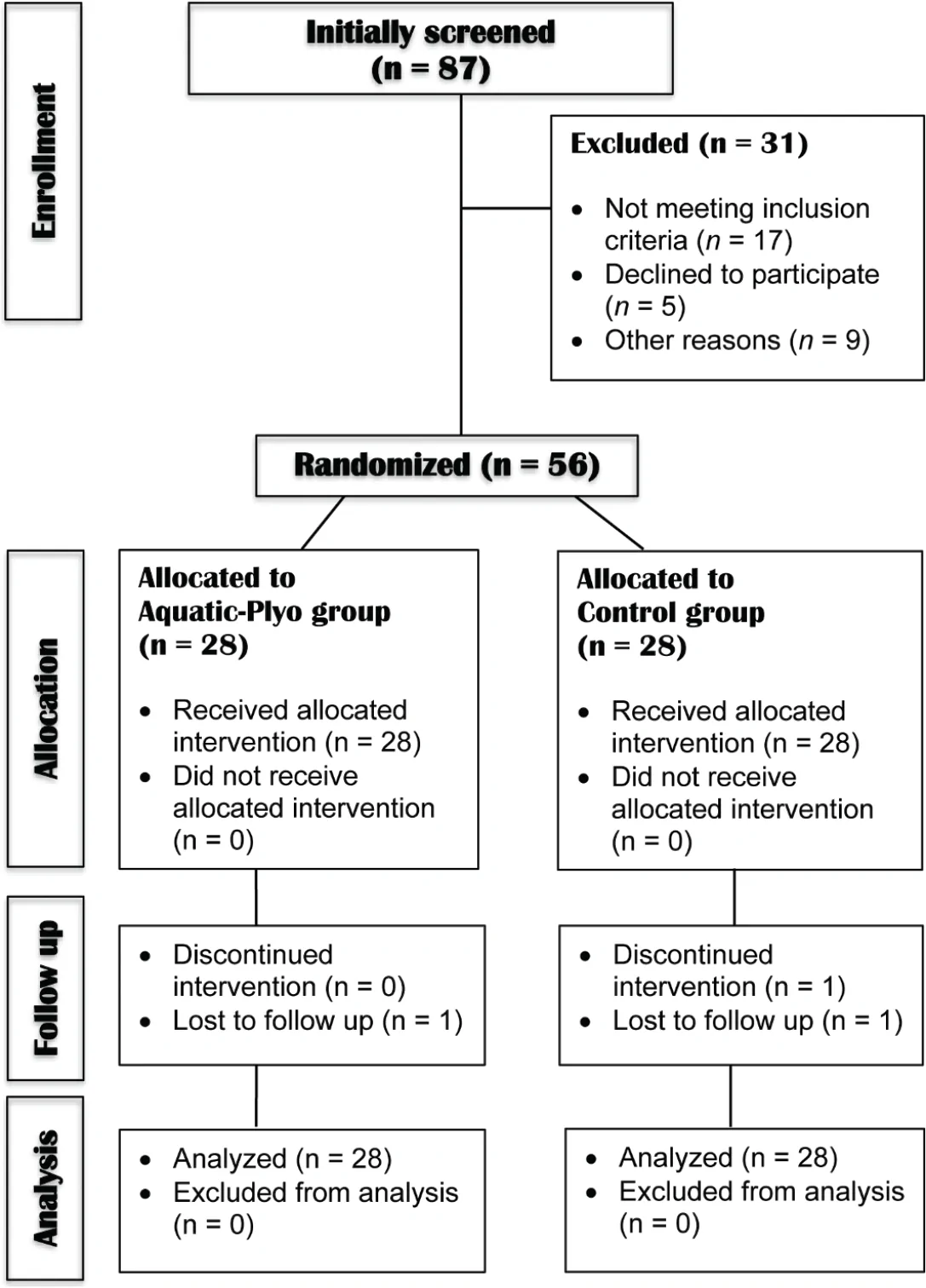
CONSORT flow diagram illustrating participant progression from initial screening and randomization through post-allocation retention, dropout, and final intention-to-treat analysis in the trial.

### Compliance with interventions

The compliance rate with the intervention, defined as the number of scheduled training sessions attended by participants out of the 24 planned over a 12-week period, was comparable across the study groups (*p* = 0.25) and indicated that non-compliance was a very marginal issue. For the Aquatic-Plyo group, the median attendance was 23 of 24 sessions (95.83%; interquartile range [IQR]: 22 to 23 sessions) compared with a median of 22.5 sessions (93.75%; IQR: 22 to 23 sessions) for the control group. The Aquatic-Plyo training was well tolerated by adolescents, who successfully completed all exercises in each session, and no adverse effects were reported.

### Baseline homogeneousness

Table I outlines the demographic, anthropometric, and PFPS-related clinical characteristics at the baseline for control and Aquatic-Plyo group participants. There was no significant difference in age, sex distribution, or anthropometric factors such as weight, height, and body mass index between the groups. Likewise, the PFPS-related clinical aspects, including pain duration, patellar tilt angle, quadriceps angle of pull, painkiller use, and engagement in physical activity, did not show significant differences across groups.

**Table I:** Participants’ baseline characteristics (demographic, anthropometric, and clinical) in the study groups

Factor	Control group (*n =* 28)	Aquatic-Plyo group (*n =* 28)	*p*-value
Age, years, mean (SD)	14.86 (1.99)	15.28 (1.76)	0.39^†^
Sex (boys/girls), *n* (%)	13 (46.4)/15 (53.6)	11 (39.3)/17 (60.7)	0.79^‡^
Weight, kg, mean (SD)	50.14 (8.20)	52.68 (8.86)	0.27^†^
Stature, m, mean (SD)	1.51 (0.10)	1.54 (0.11)	0.46^†^
Body mass index, kg/m^2^, mean (SD)	21.75 (1.47)	22.18 (1.38)	0.25^†^
Pain duration, months, mean (SD)	22.14 (6.52)	25.36 (7.73)	0.09^†^
Patellar tilt angle, degrees, mean (SD)	9.46 (2.47)	8.71 (3.99)	0.40^†^
Q-angle, degrees, mean (SD)	18.32 (2.58)	17.39 (3.64)	0.28^†^
Painkillers usage, (yes/no) *n* (%)	8 (28.6)/20 (71.4)	5 (17.9)/23 (82.1)	0.53^‡^
PA engagement, times/week, median (min–max)	3 (1–4)	3 (2–4)	0.16^¥^

† Unpaired *t*-test,

‡ χ^2^ test,

¥ ann–Whitney *U* test.

Aquatic-Plyo; aquatic plyometrics; PA: physical activity; Q-angle: quadriceps angle; SD: standard deviation.

### Between-group difference

In Fig. 2, a violin plot illustrates the differences in patient-reported pain intensity pre- and post-intervention. The median (IQR) NPRS scores for the control and Aquatic-Plyo group pre-intervention were 7 (6–9) and 6 (6–8), respectively. Post-intervention, the NPRS scores were 6 (6–7) for the control group and 4 (3–5) for the Aquatic-Plyo group. A significant large group-by-time interaction effect on pain (*F*_1__,54_ = 9.08 ; *p* = 0.004; *η*^2^p = 0.14) was identified, with the Aquatic-Plyo group reporting a greater reduction in pain intensity from the pre- to post-intervention occasion than the control group.

**Fig. 2:**
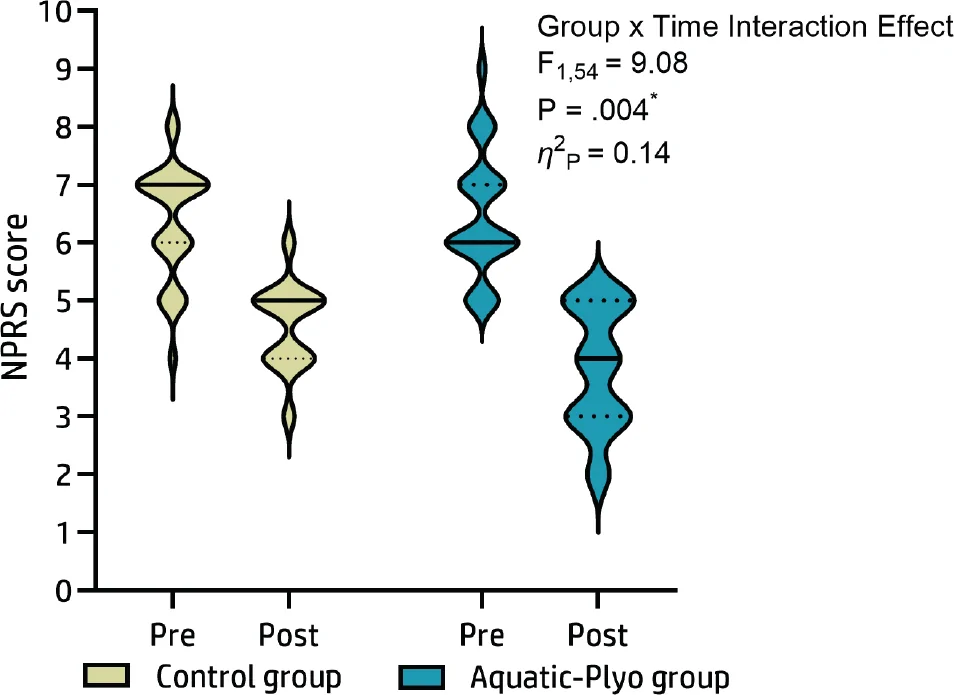
Violin plot representing patient-reported pain intensity (assessed via NPRS) at time points before and after intervention. The plot’s density illustrates score frequency in specific ranges, where wider areas signify higher frequencies and narrower areas indicate lower frequencies. Within each violin, the solid line represents the median pain score, whereas the dotted lines define the interquartile range.

Table II provides an overview of change differences in peak concentric torque for the quadriceps and hamstrings from pre- to post-intervention among the study groups. The mixed-model ANOVA uncovered a significant moderate group-by-time interaction effect on the quadriceps torque at both testing speeds of 120^o^/sec (*F*_1,54_ = 8.31; *p =* 0.006; *η*^2^p = 0.13) and 180^o^/sec (*F*_1,54_ = 7.37; *p =* 0.009; *η*^2^p = 0.12) as well as the hamstring torque at testing speeds of both 120^o^/sec (*F*_1,54_ = 7.90; *p =* 0.007; *η*^2^p = 0.13) and 180^o^/sec (*F*_1,54_ = 5.48; *p =* 0.023; *η*^2^p = 0.09). The Aquatic-Plyo group demonstrated a significant increase in the peak concentric torque for both muscle groups compared with the control group.

**Table II:** Differences in pre- to post-intervention changes in muscle strength (peak concentric torque) among the control and Aquatic-Plyo groups

Item	Control group (*n =* 28)	Aquatic-Plyo group (*n =* 28)	Group-by-time interaction	
			*p*-value	*η^2^*p
Quadriceps–Peak Concentric Torque (120^o^/sec)				
Pre	37.42 (4.72)	38.13 (5.57)	0.006^*^	0.13
Post	39.12 (5.56)	43.92 (4.68)		
*p*-value	0.036^*^	< 0.001^*^		
Hedges’ *g* (95% CI)	0.32 (0.02–0.63)	1.10 (0.57–1.66)		
Quadriceps–Peak Concentric Torque (180^o^/sec)				
Pre	31.87 (3.30)	32.40 (2.83)	0.009^*^	0.12
Post	34.10 (3.52)	36.34 (2.57)		
*p*-value	< 0.001^*^	< 0.001^*^		
Hedges’ *g* (95% CI)	0.64 (0.36–0.94)	1.42 (0.92–1.98)		
Hamstring–Peak Concentric Torque (120^o^/sec)				
Pre	29.42 (3.92)	30.36 (3.71)	0.007^*^	0.13
Post	32.12 (3.61)	35.10 (3.84)		
*p*-value	< 0.001^*^	<0.001^*^		
Hedges’ *g* (95% CI)	0.70 (0.37–1.05)	1.22 (0.84–1.66)		
Hamstring–Peak Concentric Torque (180^o^/sec)				
Pre	26.96 (3.43)	27.47 (2.88)	0.023^*^	0.09
Post	29.68 (2.79)	32.38 (3.22)		
*p*-value	< 0.001^*^	< 0.001^*^		
Hedges’ *g* (95% CI)	0.84 (0.47–1.24)	1.65 (0.93–2.27)		

Hedges’ *g*: effect size for the intra-group changes; *η*^2^*p*: effect size for the inter-group difference.

Peak concentric torque data are summarized as mean (SD).

Aquatic-Plyo: aquatic plyometrics; CI: confidence interval.

* Significant at *p* < 0.05.

Table III presents change differences in PFDI (disability) and SF-36 (HRQoL) scores from pre- to post-intervention among the study groups. A significant moderate group-by-time interaction effect on disability (*F*_1__,54_ = 4.99 ; *p =* 0.03; *η*^2^p = 0.08) and HRQoL (*F*_1,54_ = 5.49 ; *p =* 0.023; *η*^2^p = 0.09) was indicated by the mixed-model ANOVA. This analysis suggests that pre-to-post changes in disability and HRQoL were significantly different between the 2 groups, with the Aquatic-Plyo group showing a significant reduction in disability and enhancements in HRQoL relative to the control group.

**Table III:** Change differences in disability, and quality of life between the control and Aquatic-Plyo groups

Factor	Control group (*n =* 28)	Aquatic-Plyo group (*n =* 28)	Group-by-time interaction	
			*p*-value	*η*^2^p
PFDI (Disability)				
Pre	24.89 (4.78)	23.82 (5.10)	0.03^*^	0.08
Post	20.11 (3.75)	16.25 (4.22)		
*p*-value	< 0.001^*^	< 0.001^*^		
Hedges’ *g* (95% CI)	1.08 (0.71–1.50)	1.57 (0.97–2.24)		
SF-36 (HRQoL)				
Pre	75.14 (5.98)	77.11 (5.10)	0.023^*^	0.09
Post	79.21 (6.71)	84.61 (5.85)		
*p*-value	0.0004^*^	< 0.001^*^		
Hedges’ *g* (95% CI)	0.62 (0.28–0.98)	1.33 (0.83–1.88)		

Hedges’ *g*: effect size for the within-group difference; *η*^2^*p*: effect size for the between-group difference.

Disability and quality of life measures are listed as mean (SD).

Aquatic-Plyo: qquatic plyometrics; PFDI: Patellofemoral Disability Index; CI: confidence interval; SF-36: MOS 36-Item Short Form Health Survey, HRQoL: Health-Related Quality of Life.

* Significant at *p* < 0.05.

## DISCUSSION

The purpose of this study was to assess the impact of a 12-week Aquatic-Plyo training programme in adolescents with PFPS, focusing on its effectiveness on pain, muscle strength, disability, and HRQoL. The results demonstrated that, in comparison with the standard exercise therapy, the Aquatic-Plyo training led to a significant reduction in pain intensity, as shown by a substantial reduction in patient-reported NPRS scores, and an increase in muscle strength, reflected by elevated peak torque in the quadriceps and hamstrings. In terms of disability and HRQoL, the Aquatic-Plyo training was also favourably associated with a significant decrease in disability, marked by a considerable drop in the PFDI score, and an improvement in HRQoL.

Aquatic-Plyo, while not yet recognized as a mainstream exercise modality, has recently garnered interest from researchers in the field of physical rehabilitation. This is likely due to the unique therapeutic benefits that water-based plyometric activities provide, which are not observed with traditional land-based exercises (27, 28). As far as current knowledge extends, no prior research has explored the effectiveness of Aquatic-Plyo training in individuals with PFPS. However, there is some documented evidence indicating the advantages of such a training approach for both healthy children/adolescents and those suffering from a range of chronic, physically debilitating illnesses (22–26, 38, 39). Quite recently, a randomized controlled trial has explored the prospective benefits of a 12-week Aquatic-Plyo intervention for children with haemophilic knee arthropathy. The findings validated the efficacy of Aquatic-Plyo in significantly alleviating pain and achieving considerable improvement in muscle strength, postural control, and functional capabilities (22). Another up-to-date study examined the outcomes of an Aquatic-Plyo regimen in children diagnosed with juvenile idiopathic arthritis. The findings indicated that Aquatic-Plyo training effectively led to significant enhancements in muscle strength, bone mineral properties, and physical fitness levels (24). Some time back, a randomized crossover pilot study investigated the efficacy of a 4-week Aquatic-Plyo programme in children with juvenile dermatomyositis. The findings revealed that Aquatic-Plyo, in contrast to standard care, was particularly effective in fostering improvements in muscle strength, fatigue, and functional ability (26). A quasi-experimental study also assessed the efficacy of a 12-week Aquatic-Plyo regimen in mitigating the adverse physical and functional consequences in paediatric survivors of acute lymphoblastic leukaemia. The findings demonstrated considerable improvements in bone mineralization, functional performance, and HRQoL, addressing both physical and psychological aspects (23). Still, many earlier studies have explored the advantages of Aquatic-Plyo training for healthy children and adults. These studies, taken together, suggest that a 4- to 8-week regimen of Aquatic-Plyo results in notable increases in muscle power, torque, and postural control, in addition to enhancing overall physical performance (28, 38, 39). In light of the aforementioned, it is reasonable to claim that the findings demonstrated in the present study significantly bolster and align with prior studies in this field, which have associated Aquatic-Plyo training with meaningful improvements in pain, muscle strength, functional capabilities, and HRQoL, based on data from adolescents with PFPS.

Although the current study does not specifically demonstrate the mechanisms underlying the positive effects of Aquatic-Plyo, a number of plausible justifications may be relevant to the findings reported here. Plyometric-related activities have been shown to produce various neuromuscular adaptations, which include an enhanced myotatic reflex, optimized activation sequences in motor units, and improved proprioceptive awareness (40). It appears that these adaptations enhance neural firing rates and facilitate the activation of a large pool of motor neurons, which accelerate the rate of force and torque generation while improving muscle strength (24, 41–43). Moreover, plyometric workouts in this study took place in an aquatic setting, where the greater density and dynamic viscosity of water generate significantly stronger resistance to propulsive movements, creating an opposing drag force. Thus, adolescents in the Aquatic-Plyo group have been required to expend additional effort to surmount this resistance, resulting in stronger muscle development (28). In addition, the buoyancy provided by the water lessens the landing thrust experienced during Aquatic-Plyo training, allowing for a faster yet more effective transition from eccentric to concentric contractions, thereby increasing force generation (22, 24, 26, 44). The observed enhancements in muscle strength might have played a significant role in mitigating mechanical stresses and impact forces on both the knee and the surrounding muscles. This attenuation may have, in turn, alleviated patellofemoral pain, reduced associated disabilities, and enhanced functional capabilities, ultimately contributing to a better HRQoL.

A key strength of this study is its pioneering demonstration of the efficacy of Aquatic-Plyo in adolescents with PFPS. Additionally, adopting a rigorous methodology that includes a randomized controlled assessor-blinded design, the recognized gold standard for assessing the treatment effect, along with a sizeable and adequately powered sample, significantly increases the likelihood of uncovering authentic effects and offers a strong basis for drawing trustworthy conclusions regarding the intervention’s efficacy. Furthermore, the treatment focus and the collected data, which include metrics of pain intensity, muscle strength, disability, and HRQoL, directly address the key clinical issues for adolescents with PFPS, which are pertinent for their families and healthcare providers both immediately and in the long run after the intervention.

Although this study adds valuable perspectives to the research question, it is crucial to consider specific limitations to achieve a more nuanced interpretation and correct application of the findings. First, while the sample size was adequately powered for our primary objective, its relatively modest scale limits reliance on the central limit theorem to assume normality of sample means. This methodological constraint necessitated our careful selection of robust statistical frameworks, such as utilizing the non-parametric aligned ranks transformation ANOVA for the skewed ordinal pain data. Due to the nature of the intervention implemented in this study, it was not feasible to blind participants. Adolescents in the Aquatic-Plyo group may have altered their behaviour, exhibiting greater enthusiasm and increased activity levels while exercising in an appealing aquatic environment, potentially impacting the results. Therefore, a more thorough analysis is needed to explore the interaction between intervention and psychological factors. The question of whether the intervention effects last beyond the post-intervention phase remains unaddressed in this study. Thereupon, assessing the long-term effects (for instance, at 3-, 6, and 12 months later) could be worthwhile in future research. The reliance on some self-reported metrics in this study might create biases that influence the outcomes. On that account, future investigations may benefit from employing alternative strategies, such as objective evaluations, or integrating mixed-methods approaches to overcome this limitation. Finally, it is important to acknowledge that this study was conducted at a single centre, which may limit the external validity of the findings. While the physiological principles underlying Aquatic-Plyo training are universally applicable, regional variations in healthcare infrastructure, access to aquatic facilities, and sociocultural attitudes toward pool-based rehabilitation could influence patient compliance and treatment scalability in other countries. Consequently, while our findings provide a robust proof-of-concept, international multicentre trials are warranted to definitively establish the generalizability of these results across diverse global populations and healthcare systems.

The current findings provide valuable insights for clinical practice, particularly in the context of managing adolescents with PFPS. The demonstrated effectiveness of Aquatic-Plyo training in alleviating pain, enhancing muscle strength, and promoting recovery of functional capabilities supports the integration of this approach into rehabilitation protocols. Clinicians can leverage this evidence to promote a more dynamic and engaging treatment environment, which may improve patient motivation and compliance. Furthermore, the associated improvement in HRQoL suggests that Aquatic-Plyo training can address broader health outcomes, making it a holistic intervention for adolescents experiencing PFPS. As such, healthcare professionals should consider incorporating Aquatic-Plyo exercises as a standard component of care for this patient population, thereby enhancing both physical and psychological recovery.

Despite the growing body of evidence supporting the clinical superiority of aquatic interventions, their widespread integration into routine rehabilitation for PFPS remains limited. This translational gap is likely driven by the resource-demanding nature of aquatic therapy, which requires access to specialized pool facilities, rigorous maintenance protocols, and healthcare professionals trained in hydrotherapy. For standard outpatient physical therapy clinics, the financial and logistical burdens of maintaining an aquatic facility may be prohibitive. Therefore, the decision to adopt Aquatic-Plyo training may frequently depend on cost-effectiveness rather than clinical efficacy alone. To overcome these barriers and facilitate implementation, clinics might explore strategic partnerships with local community aquatic centres or public pools. Furthermore, centralizing such specialized programmes within larger hospital-based rehabilitation networks could optimize resource allocation. Ultimately, future research must incorporate rigorous cost-effectiveness analyses to determine whether the higher initial operational costs of Aquatic-Plyo training are offset by accelerated recovery times, reduced injury recurrence, and profound long-term enhancements in HRQoL.

In conclusion, the findings of this study present compelling evidence that Aquatic-Plyo training is an effective therapeutic intervention for alleviating pain, enhancing muscle strength, decreasing disability, and improving HRQoL in adolescents with PFPS. Such evidence supports the inclusion of Aquatic-Plyo exercises in rehabilitation protocols for such a patient population. To further contextualize these findings, it would be advantageous to conduct research aimed at uncovering the potential mechanisms through which the Aquatic-Plyo training influences these outcomes, which could inform better exercise prescription strategies.

## Supplementary Material


