# Supplementary material for: CLINICAL AND FUNCTIONAL OUTCOMES OF AQUATIC PLYOMETRICS IN ADOLESCENT PATELLOFEMORAL PAIN SYNDROME: A DUAL-ARM RANDOMIZED CONTROLLED TRIAL
Source: J Rehabil Med. 2026 Oct 19;58:46495. doi: 10.2340/jrm.v58.46495 (PMC13685587; doi:10.2340/jrm.v58.46495)
Supplement: Supplementary file 1 [file JRM-58-46495-s1.pdf]

Table SI. Specifics of the Aquatic-Plyo exercises, performance instructions, progression pace, and training volume

Exercise	Description and instruction for performance	W 1 & 4	W 5 & 8	W 9 & 12
Ankle hops	Stand with feet at the shoulder-width, keep legs straight and arms by side, hop up with movement primarily at ankle joints, repeat promptly on landing	3/10	3/15	5/10
Single-leg hops	Stand on one leg, with the other leg pointing behind the knee, buttocks back, and chest forward. Perform ¼ a squat, then push through the floor explosively to allow the body to hop from the ground.	1/10 RT<br>1/10 LT	1/15 RT<br>1/15 LT	2/10 RT<br>2/10 LT
Tuck jump	Stand with feet shoulder-width apart and chest up, drop down a little into a ½ squat, drive your arms up and push off the bottom of the pool, tuck knees toward your chest as much as possible, bend the knees on landing to absorb the impact.	1/10	2/15	3/10
Countermovement jump	Stand upright with feet at the shoulder-width, perform a preliminary downward movement by flexing at the knees and hips, then explosively jump up as high as possible.	2/5	3/5	4/5
Lateral jump	Stand with feet hip-width apart, bend knees to squat straight down, quickly push upward and sideways (RT and LT side alternately), land softly, and absorb the shock by squatting deeply	1/8	2/8	3/8
Standing long jump	Standing with a straight back, bend the knees and push the hips back, swing arms forward, jump and launch forward to the maximum extent, and land in a squat position. Then, return to starting position and repeat	1/5	2/5	3/5

Drop jump	Stand with feet shoulder-width apart on a 10-inch-high step, step slightly forward off the step, land toward the forefeet without any upward movement, react as quickly as possible to the floor of the pool, and spring immediately back up.	1/5	2/5	3/5
Box jump	Stand with feet shoulder-width apart, 20-cm away from a 10-inch-heigh step, bend knees slightly and drop down, bringing arms out behind, jump onto the box, and land softly on both feet with a slight bend in the knees, then step back and down and repeat	2/3	3/3	4/3
Single-leg vertical jump	Stand on one leg, bend hips and knees down, then jump up explosively to the maximum height, and land softly on both feet with a slight bend in the knees.	1/4 RT<br>1/4 LT	2/4 RT<br>2/4 LT	3/4 RT<br>3/4 LT
Single-leg lateral jump	Stand on one leg, bend hips and knees down, then jump laterally (alternately between the RT and LT side) as far as possible, and land softly on both feet with a slight bend in the knees.	1/5 RT<br>1/5 LT	2/5 RT<br>2/5 LT	3/5 RT<br>3/5 LT

- W: week, RT: right side, LT: left side.
- Exercise volume presented as the number of sets/repetitions; single-leg exercises were repeated on the other side with similar set/repetition numbers.
- Exercise repetitions were performed successively with no pauses. Intervals of rest for 1-2 minutes were given between exercise sets.
- Participants were encouraged to exert as maximal effort as they can while performing each exercise.
- Training volume/intensity of the first block (week 1 & 2) was determined through a preliminary test of children’s performance (five children)
